# Preparation and characterization of pH sensitive crosslinked Linseed polysaccharides-co-acrylic acid/methacrylic acid hydrogels for controlled delivery of ketoprofen

**DOI:** 10.1080/15685551.2017.1368116

**Published:** 2017-09-13

**Authors:** Farya Shabir, Alia Erum, Ume Ruqia Tulain, Muhammad Ajaz Hussain, Mahmood Ahmad, Faiza Akhter

**Affiliations:** ^a^ Faculty of Pharmacy, University of Sargodha, Sargodha, Pakistan; ^b^ Ibn-e-Sina Block, Department of Chemistry, University of Sargodha, Sargodha, Pakistan; ^c^ Faculty of Pharmacy and Alternative Medicine, The Islamia University of Bahawalpur, Bahawalpur, Pakistan

**Keywords:** pH responsive, hydrogel, Linseed hydrogel-co-acrylic acid, Linseed hydrogel-co-methacrylic acid, ketoprofen, drug release

## Abstract

Some pH responsive polymeric matrix of Linseed (*Linum usitatissimum*), L. hydrogel (LSH) was prepared by free radical polymerization using potassium persulfate (KPS) as an initiator, *N,N*-methylene bisacrylamide (MBA) as a crosslinker, acrylic acid (AA) and methacrylic acid (MAA) as monomers; while ketoprofen was used as a model drug. Different formulations of LSH-co-AA and LSH-co-MAA were formulated by varying the concentration of crosslinker and monomers. Structures obtained were thoroughly characterized using Fourier transforms infrared (FTIR) spectroscopy, XRD analysis and Scanning electron microscopy. Sol-gel fractions, porosity of the materials and ketoprofen loading capacity were also measured. Swelling and *in vitro* drug release studies were conducted at simulated gastric fluids, i.e., pH 1.2 and 7.4. FTIR evaluation confirmed successful grafting of AA and MAA to LSH backbone. XRD studies showed retention of crystalline structure of ketoprofen in LSH-co-AA and its amorphous dispersion in LSH-co-MAA. Gel content was increased by increasing MBA and monomer content; whereas porosity of hydrogel was increased by increasing monomer concentration and decreased by increasing MBA content. Swelling of copolymer hydrogels was high at pH 7.4 and low at pH 1.2. Ketoprofen release showed an increasing trend by increasing monomer content; however it was decreased with increasing MBA content. Sustained release of ketoprofen was noted from copolymers and release followed Korsmeyer-Peppas model.

## 1. Introduction

In order to deliver a drug through the oral route, it is important to consider physiological pH of the gastrointestinal tract [1–3]. The abrupt physiological pH changes in human body may cause severe problem in the delivery of sensitive drugs. So, there is dire need to develop new and improved drug delivery methods to overcome such problems. Delayed release drug delivery systems and enteric coating techniques appeared as valuable tools for effective drug delivery in all physiological pH environments.[4] Among delayed and targeted drug release systems, stimuli sensitive hydrogel based formulation design appeared highly valuable to deliver drugs directly to colon after by passing the acidic stomach environment [1,5–9].

Hydrogels isolated from plant materials are mainly cross-linked polysaccharides that absorb high amounts of water and generally swells at intestinal pH and shrink at stomach’s pH [5]. High swelling index, cross-linked network structure, stimuli responsive nature make water swellable polysaccharides as smart materials or intelligent drug delivery system. Such polysaccharides generally have ionic pendant groups able to accept and/or donate protons as a response to change in physiological pH which is an integral factor of pH responsive hydrogels [6,9–11]. A recent study has evaluated the Linseed hydrogel (LSH) as a stimuli responsive hydrogel that have very high swelling capacity and offers pH sensitive swelling-shrinking properties [5,12].

By grafting the natural polysaccharides with synthetic polymers, one may get benefit of the properties of both entities. On this concept, various polysaccharides of natural origin have been modified by different monomers. Grafting of acrylic acid and methacrylic acid as monomers on to such polysaccharides by free radical polymerization produced effective pH responsive hydrogels [13–17].

Herein, attempt was made to copolymerize LSH with AA and MAA monomers. Obtained novel structures were characterized. Aims are to evaluate LSH-co-AA and LSH-co-MAA for pH responsive delivery of ketoprofen.

## 2. Experimental

### 2.1. Materials

Seeds of *Linum usitatissimum* (Linseed) were purchased from local market of Sargodha, Pakistan. Acrylic acid (AA), methacrylic acid (MAA), potassium persulfate (KPS), NaOH and potassium dihydrogen phosphate were purchased from Sigma Aldrich, Germany. *N,N*-methylene bisacrylamide (MBA) was obtained from Fluka, Switzerland. HCl, ethanol and *n*-hexane were procured from Riedel-de Haën, Germany. Ketoprofen was received as a gift from Danas Pharmaceuticals, Islamabad, Pakistan.

### 2.2. Extraction of LSH

LSH was isolated as per reported method [5]. Briefly, after manual cleaning and screening, seeds were soaked deionezed water for 24 h at room temperature. After 24 h soaked seeds were heated at 80 °C for 30 min. The extracted mucilage was separated from seeds using muslin cloth and was treated with sufficient quantity of *n*-hexane for removal of non-polar material like waxes. Washed mucilage (LSH) was dried at 60 °C and preserved in air tight containers after grinding. Characterization of LSH hence obtained, has already been reported in literature [5,12,18].

### 2.3. Synthesis of LSH copolymers

LSH was suspended in deionozed water (1% w/v) with continuous stirring at 70 °C. KPS was added in to it. A solution of MBA was separately prepared in acrylic acid (monomer) and added to polymer-initiator solution. Reaction mixture was kept firstly at 60 °C for 30 min then temperature was raised to 80 °C up to 24 h. After 24 h, transparent hydrogels were formed, which were then washed with (30% ethanol: water) to eliminate any non-reacted monomer and surplus reagents. Copolymers were dried at 50 °C and stored in air tight jars for further use in characterization. LSH-co-MAA hydrogels were prepared by same method as of LSH-co-AA, except that instead of acrylic acid, MAA was used [16,19]. Composition for LSH-co-AA and LSH-co-MAA formulations is given in Tables 1 and 2, respectively.

**Table 1.** Composition for LSH-co-AA hydrogels/100 g.

Formulation code	LSH (g/100 g)	Acrylic acid (g/100 g)	Initiator (g/100 g)	Crosslinker (g/100 g)
A1	1.0	15	0.2	0.3
A2	1.0	15	0.2	0.4
A3	1.0	15	0.2	0.5
A4	1.0	12.5	0.2	0.2
A5	1.0	15	0.2	0.2
A6	1.0	17.5	0.2	0.2

**Table 2.** Composition for LSH-co-MAA hydrogels/100 g.

Formulation code	LSH (g/100 g)	MAA (g/100 g)	Initiator (g/100 g)	Crosslinker (g/100 g)
M1	2.5	25	0.2	0.3
M2	2.5	25	0.2	0.4
M3	2.5	25	0.2	0.5
M4	2.5	27.5	0.2	0.2
M5	2.5	30	0.2	0.2
M6	2.5	35	0.2	0.2

### 2.4. Swelling studies

To optimize and determine pH sensitivity of the prepared formulations, swelling studies were conducted at pH 1.2 and 7.4. Both swelling ratio (*q*) and percentage equilibrium swelling (% ES) were measured.

(1) $$q=M_{S}/M_{o}$$

where, *M*_*S*_ is the mass of swollen hydrogel at time *t* and *M*_*o*_ is the mass of dry hydrogel disc.

(2) $$\%\;\text{ES}=\left(M_{\text{eq}}-M_{o}\right)/M_{\text{eq}}\times 100$$

where, *M*_eq_ is swollen gel mass at time of equilibrium, *M*_*o*_ shows dried mass of disc. Hence, % ES was calculated [20].

### 2.5. FTIR spectroscopy

Fourier transforms infrared (FTIR)-spectra of pure drug, hydrogel and drug loaded hydrogel were recorded on IR prpestige-21 (Shimadzu, Japan). For samples analysis, KBr tablets of samples were prepared under 150 kg/cm^2^ hydraulic pressure. Samples (glassy discs) were then scanned over the range of wave number 4000–500 cm^−1^ at room temperature [19].

### 2.6. Scanning electron microscopic analysis

Hydrogel surface morphology was determined using scanning electron microscope (FEI, Quanta 400). For sample preparation hydrogel discs were placed on carbon stub. After fixing samples on stub hydrogel discs were examined under electron microscope [15].

### 2.7. X-rays diffraction analysis

To determine changes in physical form of drug X-ray diffraction (XRD) spectrum of pure drug and drug loaded and unloaded polymeric matrix was conducted. The XRD was performed at the angle between 0° and 50° and scan rate was set at 1° min^−1^ at 2*θ* using Panalytical differential scanning calorimeter [16].

### 2.8. Determination of sol–gel fraction

Hydrogel disc of all preparations were soaked in distilled water for 48 h at room temperature. The samples were prepared at a dilute concentration (typically ~1%) to ensure that hydrogel material is fully dispersed in water. The gel fraction was then measured as follows:

(3) $$\text{Gel fraction}\;\left(\text{hydrogel}\;\%\right)=\left(W_{d}/W_{i}\right)\times 100$$

(4) Sol fraction=100-Gel Fraction

where, *W*_*i*_ is the initial weight of dried sample and *W*_*d*_ is the weight of the dried insoluble part of sample after extraction with water [21,22].

### 2.9. Porosity measurement

Solvent replacement method was used to determine porosity. Dried hydrogels were immersed in ethanol overnight and weighed after excess ethanol on the surface was blotted. The porosity was calculated from the following equation [23].

(5) $$\text{Porosity}=(M_{2}-M_{1})/\rho V\times 100$$

where, *M*_1_ and *M*_2_ are the mass of hydrogel before and after immersion in ethanol respectively, *ρ* is the density of absolute ethanol and *V* is the volume of the hydrogel [20].

### 2.10. Drug loading onto copolymers

Each formulation disc was soaked in 50 mL of 1% w/v drug solution after weighing for a period of 48 h at ambient temperature. After 48 h, discs were removed from drug solution, washed with distilled water and dried at 50 °C for 2–3 days [24].

### 2.11. Drug loading determination

Loaded drug amount was calculated by weight and extraction method. A dried disc of every formulation was dipped in 1% w/v drug solution after weighing. After 48 h, swelling and drying, weight of loaded disc was taken and initial weight of unloaded disc was excluded from it to determine loaded drug.

(6) $$\text{Amount of drug}=W_{D}-W_{d}$$

(7) $$\text{Drug loading}\;\left(\%\right)=\left[\left(W_{D}-W_{d}\right)/W_{d}\right]\times 100$$

where, *W*_*d*_ and *W*_*D*_ are the weight of dried hydrogels before and after immersion in drug solution, respectively.

In extraction method, repeated extraction of the weighted quantity of loaded hydrogels was done by using deionized water. Each time 25 mL fresh 50% deionized water solution was used until there was no drug in the solution. Drug concentration was determined spectrophotometrically at *λ*_max_ 255 nm with a molar extinction coefficient of 15,508.5 L mol^−1^ cm^−1^. Amount of drug present in all portions was considered as total amount of drug loaded onto hydrogel [25].

### 2.12. In vitro drug release

Dissolution studies were performed using USP-dissolution apparatus II (Pharma test, Germany) at 37 ± 0.5 °C. Each gel disc was firstly placed in HCl buffer for 2 h, then same disc was shifted in buffer medium and release was observed for 24 h at 50 rpm at *λ*_max_ 255 nm. About 5 mL of sample was removed for the sake of analysis. It was replaced with fresh medium [24]. To determine drug release, the formula used was:

(8) $$\%\;\text{drug release}=F_{t}/F_{\text{load}}\times 100$$

where, *F*_*t*_ shows the quantity of ketoprofen released at any time *t* and *F*_load_ represents the quantity of ketoprofen that was loaded in hydrogel matrix. Dissolution data modelling was done by DD solver software of data analysis [15].

## 3. Results and discussion

### 3.1. Synthesis of LSH copolymers

After many trials, it was found that the optimum concentration of LSH for LSH-co-AA and LSH-co-MAA formulations was 1 and 2.5% respectively.

### 3.2. Swelling studies

**Figure 1.**
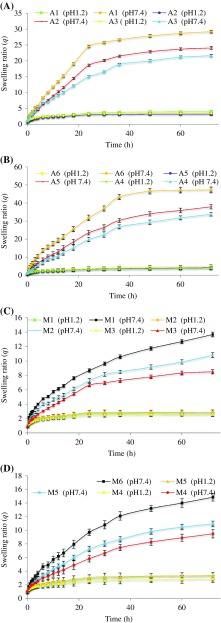
Comparative swelling ratios of LSH-co-AA formulations (A) varying MBA content (B) varying AA content, &LSH-co-MAA formulations (C) varying MBA content (D) varying MAA content at pH 1.2 and 7.4.

**Figure 2.**
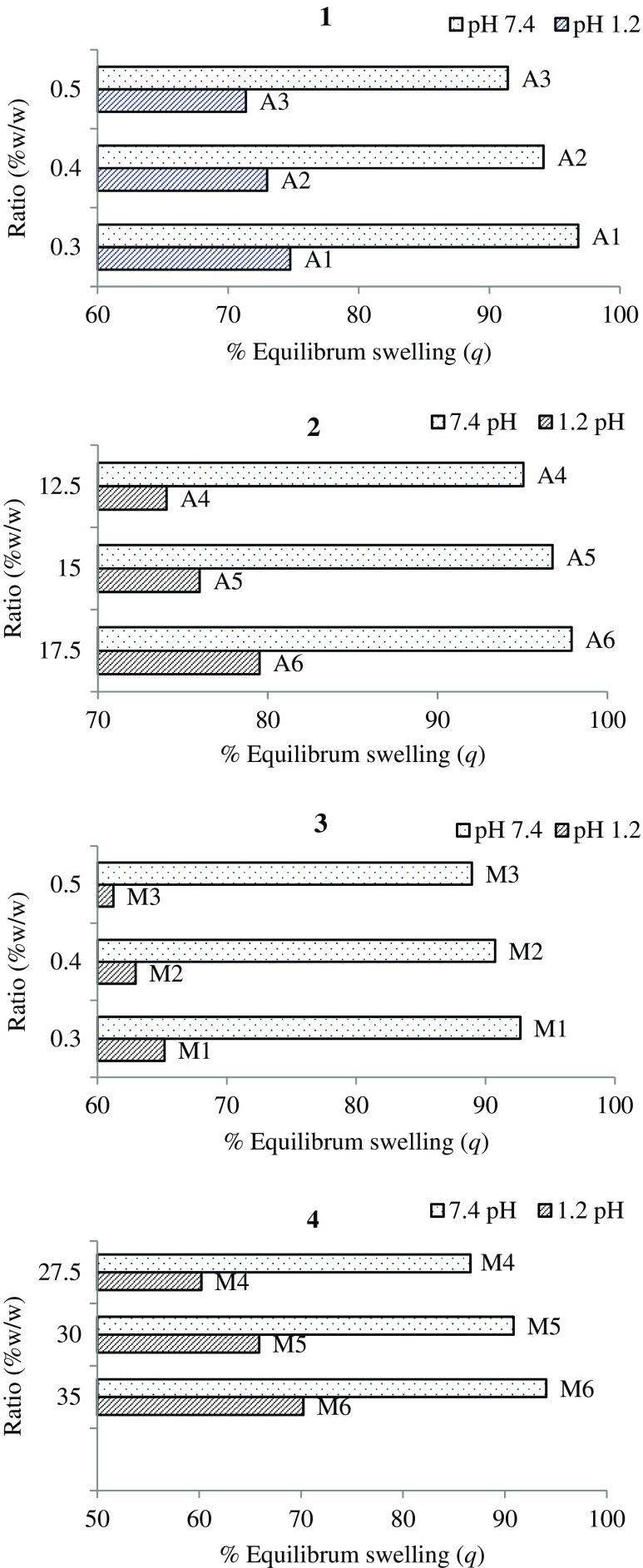
%ES of LSH-co-AA formulations (1) varying MBA content (2) varying AA content, &LSH-co-MAA formulations (3) varying MBA content (4) varying MAA content at pH 1.2 and 7.4.

Swelling studies (dynamic swelling) of copolymer were conducted on all formulations for a period of 72 h at pH 1.2 and pH 7.4. Swelling ratio (*q*, i.e., dynamic swelling) and equilibrium swelling (%ES) were carefully determined (Figures 1 and 2, respectively). All formulations (A1–A6 and M1–M6) showed stimuli responsive swelling behaviour which can be observed by swelling of copolymers at pH 1.2 and 7.4. At low pH value, most carboxylic acid groups of hydrogel were in the form of COOH, hence less swelling was observed. As the environmental pH value rose to 7.4, (1) carboxylic acid groups began to ionize, (2) osmotic pressure inside the hydrogels was increased and (3) electrostatic repulsion causes the network to expand [15]. Figure 1 showed that as the concentration of crosslinker increased, a decrease in swelling ratio was observed with A1, A2 & A3 at pH 7.4. This fact is witnessed by literature, as concentration of crosslinker increases, crosslink points also increases which results in high crosslink density thus decreasing water absorbency of hydrogel [26–28].

The reason behind higher swelling of A4–A6 formulations at pH 7.4 as compared to pH 1.2 is that as AA contents increases, an electrostatic repulsive force operating between the charged carboxyl groups of acrylic acid increases which increases hydration of the hydrogels, causing swelling [13–15,19,29,30].

As the nature of MAA and AA is same therefore similar trends in dynamic and equilibrium swelling of LSH-co-AA hydrogel formulations were observed (see Figures 1 and 2). This copolymer thus appeared pH responsive, as well, as it swells at pH 7.4 while showed less swelling at pH 1.2. Likewise, LSH-co-MAA showed comparably high swelling at pH 7.4 for the formulation having less concentrations of crosslinker.

Swelling ratios of LSH-co-AA appeared higher than LSH-co-MAA because of the presence of an additional hydrophobic methyl group in LSH-co-MAA formulations as compared to LSH-co-AA hydrogels [31].

### 3.3. FTIR analysis

**Figure 3.**
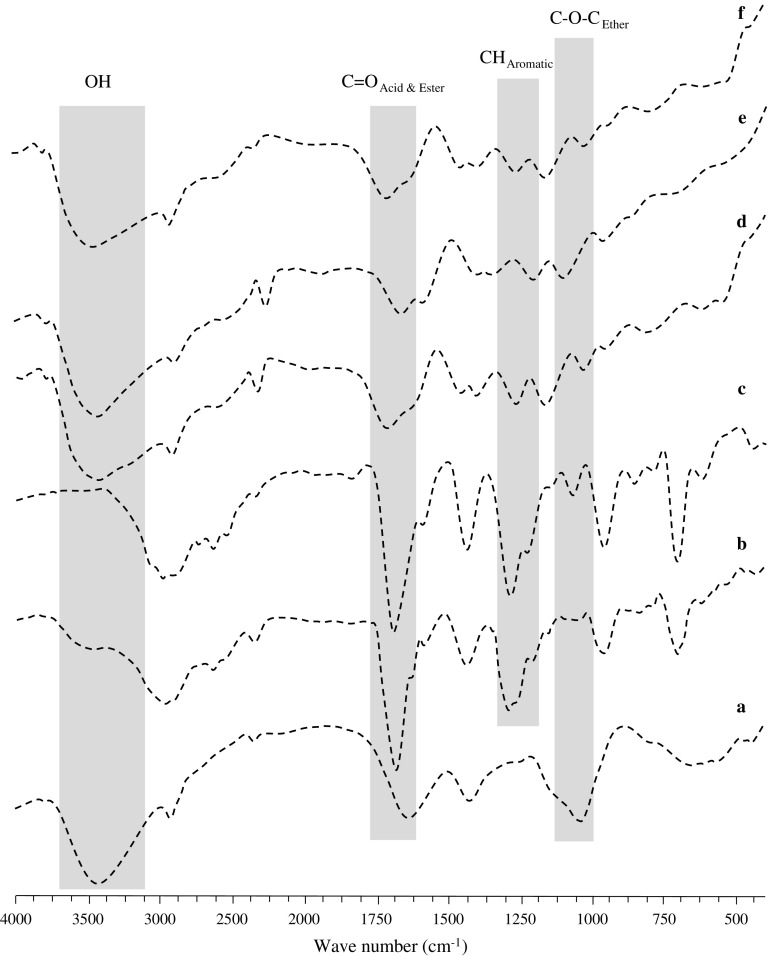
FTIR overlays of; (a) LSH, (b) ketoprofen, (c) ketoprofen loaded LSH-co-AA formulation (d) ketoprofen loaded LSH-co-MAA formulation, (e) LSH-co-AA and (f) LSH-co-MAA.

FTIR spectra of LSH, ketoprofen, ketoprofen loaded LSH-co-AA, ketoprofen loaded LSH-co-MAA, LSH-co-AA and LSH-co-MAA was recorded using KBr pellet method. LSH showed absorption at 3433 cm^−1^ (OH stretching), 2870 cm^−1^ (aliphatic CH stretching) and 1722 cm^−1^ (carboxylic acid C=O stretching) [Figure 3(a)] which was probably due to acidic fractions (type I rhamnogalacturonans) present in LSH [18]. Whereas, ketoprofen spectrum [Figure 3(b)] showed absorption bands at 1684 cm^−1^ represent C=O of COOH group and 1437 cm^−1^ represents aromatic C–H stretching [32].

FTIR spectroscopic analysis showed that no degradation of any formulation was observed as all vital signals of the polymers and drug are present in FTIR spectra of ketoprofen loaded LSH-co-AA and LSH-co-MAA [see Figure 3(c) and (d)]. FTIR spectra also revealed the grafting of AA and MAA monomers onto LSH due to absence of their carboxylic acid carbonyls absorptions at 1759 and 1739 cm^−1^, respectively [13,16] while appearance of 1722 cm^−1^ indicates grafting to the LSH [see Figure 3(e) and (f)].

### 3.4. Scanning electron microscopy

**Figure 4.**
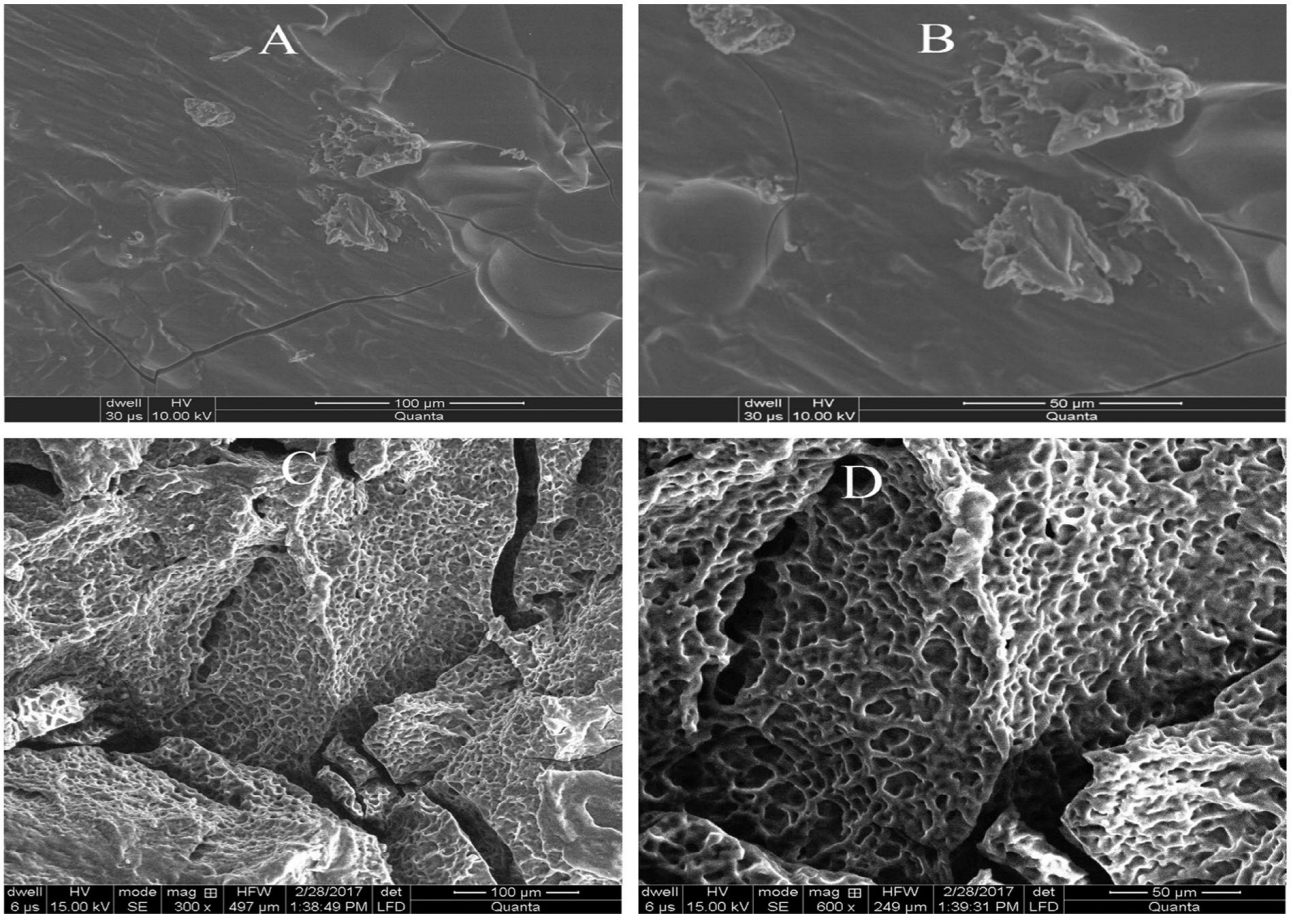
SEM images of LSH-co-MAA (A, B) and LSH-co-AA (C, D).

For determining morphological characteristics of prepared hydrogels, scanning electron microscopy was performed for formulations of LSH-co-AA and LSH-co-MAA with best swelling results. Scanning electron microscopy (SEM) images given in Figure 4 depicted porous morphology for LSH-co-AA hydrogel while micro to nano cracks were recorded onto LSH-co-MAA hydrogel.

### 3.5. Powder X-rays diffraction analysis

**Figure 5.**
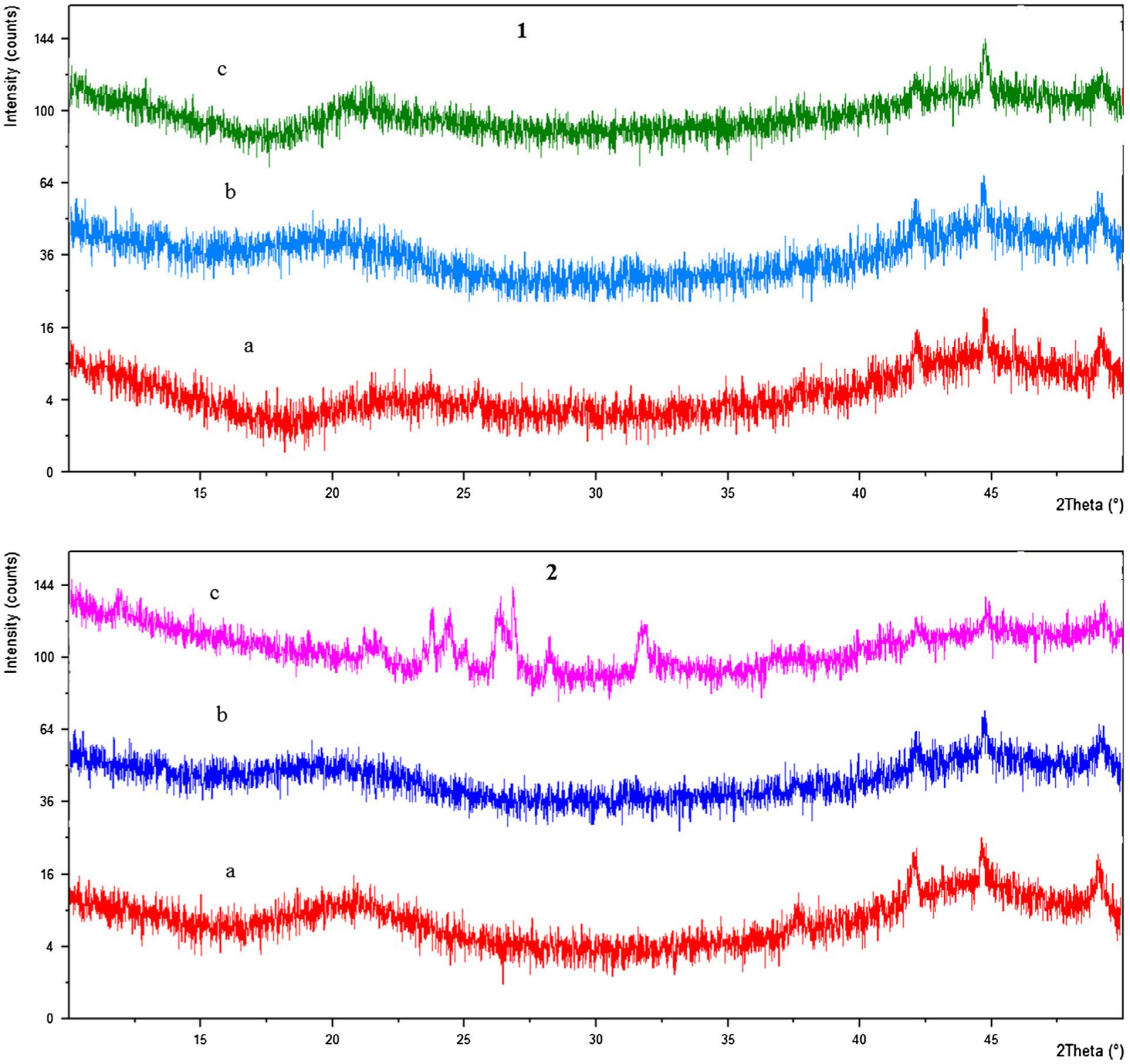
Overlaid XRD spectra of; [1] (a) unloaded LSH-co-AA (b) ketoprofen (c) ketoprofen loaded LSH-co-AA and [2] (a) unloaded LSH-co-MAA (b) ketoprofen (c) ketoprofen loaded LSH-co-MAA.

Powder X-rays diffraction (PXRD) analyses of drug loaded samples and drug was performed and it was determined that crystalline nature of drug retained after its entrapment in dosage form. Overlaid PXRD graphs of ketoprofen, unloaded and ketoprofen loaded LSH-co-AA and LSH-co-MAA are given in Figure 5.

PXRD analyses have revealed that characteristic peaks of ketoprofen in LSH-co-AA spectra with almost no significant difference in intensity of ketoprofen characteristic peaks. While in case of LSH-co-MAA overlay, characteristic peaks of ketoprofen showed a clear decrease in its intensity. Therefore, results have indicated that in LSH-co-AA disc, ketoprofen retained its crystalline form and in LSH-co-MAA, ketoprofen underwent amorphous dispersion [32].

### 3.6. Determination of sol-gel fraction/percent gel content

**Figure 6.**
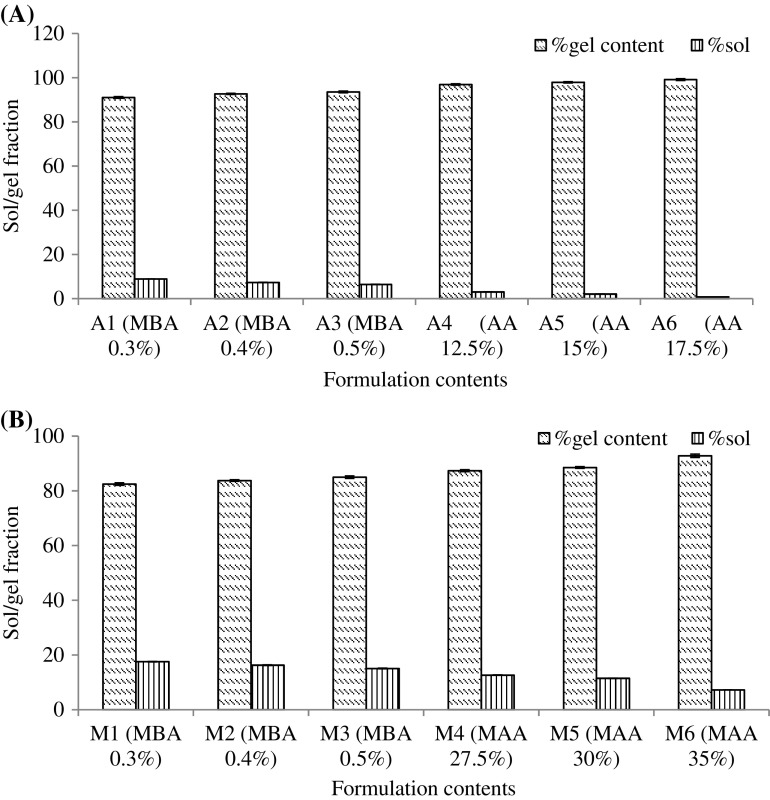
Percent sol-gel content of (A) LSH-co-AA and (B) LSH-co-MAA hydrogels.

Sol-gel fraction/percent gel content of all LSH-co-AA and LSH-co-MAA formulations is given in Figure 6.

In all LSH-co-AA formulations, gel fraction increased and sol fraction was decreased with increasing MBA and monomers content. Same was observed for LSH-co-MAA formulations. Reason for increase gel fraction is that, with increasing monomer and crossslinker concentration there will be more crosslinking which will ultimately increase the gel strength and hence gel fraction [20].

### 3.7. Porosity measurement

**Figure 7.**
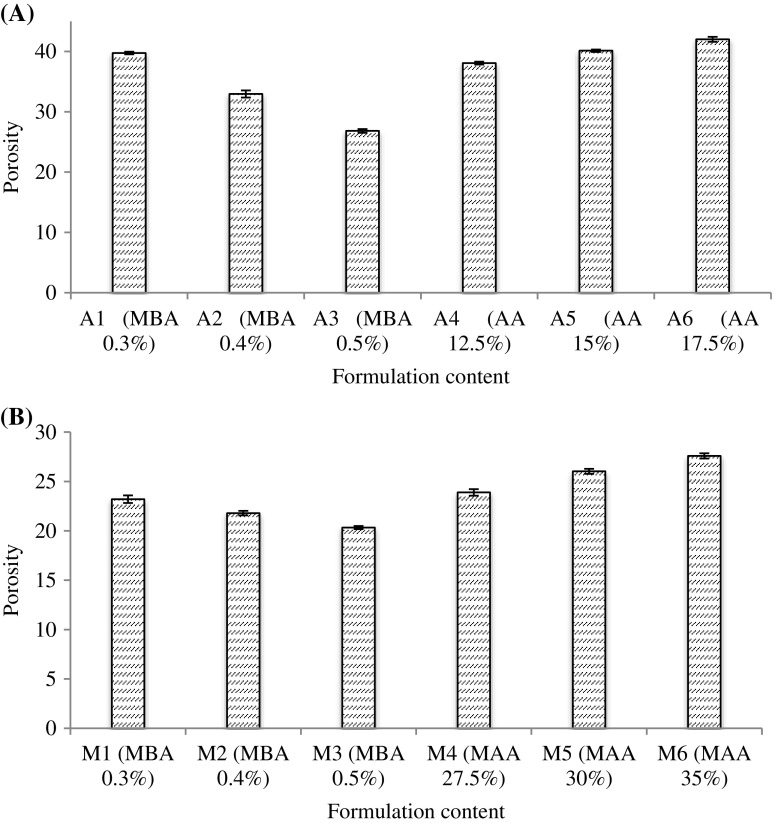
Porosity measurement of (A) LSH-co-AA and (B) LSH-co-MAA formulations.

Porosity measurement of all LSH-co-AA and LSH-co-MAA formulations is given in Figure 7. According to results, LSH-co-AA formulations with varying MBA content showed a decrease in porosity with increasing MBA content and an increase in porosity was observed with increasing monomer content. Same was observed with LSH-co-MAA formulations. This can be explained as by increasing the concentration of monomer; the viscosity of solution increased which prevented the bubbles to escape from solution forming interconnected channels thus porosity increased. By increasing the concentration MBA porosity was decreased. As molecular entanglement between polymer and monomer increased by increasing crosslinking density, there was a decrease in mesh size of hydrogen and less pore formation which resulted in decreased porosity [20,25].

### 3.8. Determination of drug loading capacity

Drug loading capacity of LSH-co-AA &LSH-co-MAA is given in Table 3.

**Table 3.** Drug loading capacity in all formulations of LSH-co-AA and LSH-co-MAA.

Formulation code	Ketoprofen (mg/g disc)	
	Weight method mean ± S.E.M	Extraction method mean ± S.E.M
A1	426.4 ± 2.3	419.3 ± 2.1
A2	390.3 ± 1.7	385.2 ± 1.5
A3	372.5 ± 2.2	367.1 ± 1.3
A4	509.5 ± 1.4	499.5 ± 2.5
A5	578.7 ± 1.2	559.7 ± 3.1
A6	676.5 ± 3.0	668.3 ± 3.3
M1	249.2 ± 1.4	245.4 ± 1.3
M2	174.3 ± 0.9	163.5 ± 0.9
M3	149.1 ± 1.2	142.8 ± 1.1
M4	165.9 ± 1.5	159.8 ± 1.4
M5	267.4 ± 1.3	260.5 ± 1.3
M6	342.3 ± 1.9	333.7 ± 2.0

### 3.9. In vitro drug release measurement

**Figure 8.**
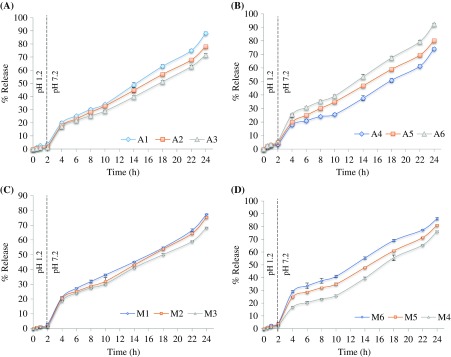
*In vitro* release profile of LSH-co-AA formulations (a) varying MBA content (b) varying AA content, &LSH-co-MAA formulations(c) varying MBA content (d) varying MAA content at pH 1.2 and 7.4.

*In vitro* release profiles of LSH-co-AA and LSH-co-MAA hydrogels with varying crosslinker concentration and varying monomer concentration at pH 1.2 and 7.4 are shown in Figure 8. All formulations showed a pH dependent release with less than 6% ketoprofen release in first 2 h at pH 1.2. The cumulative percentage release of ketoprofen from the hydrogels was lower at pH 1.2 than pH 7.4, which was mainly due to lower swelling of hydrogel in acidic environment.[33] Formulations with varying MBA content, showed a decrease in drug release with increasing MBA content. As for A1, A2 and A3, ketoprofen release after 24 h was 88.19, 78.09 and 71.60%, respectively. Formulations with varying AA content showed an increase in drug release with increasing AA content. As for A4, A5 and A6, ketoprofen release after 24 h was 74.08, 80.19 and 92.39%, respectively. Among all LSH-co-AA formulations, the A6 showed highest release at pH 7.4, which was expected due to its greater AA contents resulting in maximum hydration and swelling and more pH sensitivity [15,24]. Like LSH-co-AA formulations, LSH-co-MAA formulations also showed a decrease in drug release with increasing MBA content. As for M1, M2 and M3, percentage ketoprofen release after 24 h was 77.36, 75.34 and 68.35%, respectively. Formulations with varying MAA content showed an increase in drug release with increasing MAA content. As for M4, M5 and M6, ketoprofen release after 24 h was 76.12, 80.96 and 86.12%, respectively.

This significant drug release was observed due to ionization of carboxylic acid groups of MAA. Ionization of carboxylic group enhanced the swelling of hydrogel as well as drug release. While increase in crosslinking agent, decreased the swelling ratio and interaction of drug molecules with the physiological medium and hence decreased the drug release [16].

### 3.10. Evaluation of drug release kinetics

For assessment of drug release kinetics, various kinetic models like Zero order, First order, Higuchi, Hixson-Crowell and Korsmeyer-Peppas models were applied by using DD Solver software. Values of correlation coefficient (*R* ^2^) and release rates of all formulations are mentioned in Tables 4 and 5. From values of *R* ^2^ of trend line of graph, it can be seen that Korsmeyer-Peppas model best fits to all formulations. This model is applied to determine the release mechanism of drug, i.e., Fickian diffusion or non-Fickian diffusion. Values of *n* for all preparations in this study were greater than 0.7 and less than 1 showing non-Fickian diffusion [34].

**Table 4.** Correlation coefficient (R^2^) of LSH-co-AA &LSH-co-MAA formulations.

Formulation code	Release model				
	Zero order	First order	Higuchi	Korsmeyer-Peppas	Hixson-Crowell
A1	0.9914	0.8716	0.9576	0.9919	0.9746
A2	0.9903	0.9716	0.8742	0.9914	0.9831
A3	0.9876	0.9716	0.8696	0.9884	0.9810
A4	0.9841	0.9582	0.8583	0.9854	0.9700
A5	0.9878	0.9782	0.8980	0.9935	0.9879
A6	0.9799	0.9647	0.9026	0.9887	0.9784
M1	0.9706	0.9772	0.9024	0.9828	0.9814
M2	0.9774	0.9727	0.8869	0.9829	0.9795
M3	0.9731	0.9797	0.8954	0.9824	0.9813
M4	0.9887	0.9537	0.8435	0.9895	0.9688
M5	0.9721	0.9725	0.9011	0.9833	0.9798
M6	0.9570	0.9766	0.9196	0.9818	0.9801

**Table 5.** Release rates of all LSH-co-AA &LSH-co-MAA formulations.

Formulation code	Release model					
	Zero order	First order	Higuchi	Korsmeyer-Peppas		Hixson-Crowell
	*K*_O_ (h^−1^)	*K*_1_ (h^−1^)	kH (h^−1^)	kKP (h^−1^)	*n*	kHC (h^−1^)
A1	3.558	0.054	14.045	3.979	0.961	0.016
A2	3.211	0.047	12.681	3.767	0.944	0.013
A3	2.928	0.041	11.560	3.350	0.953	0.012
A4	2.901	0.040	11.433	2.970	0.991	0.012
A5	3.333	0.050	13.26	4.714	0.880	0.014
A6	3.834	0.063	15.302	5.856	0.853	0.018
M1	3.226	0.048	12.899	5.244	0.832	0.014
M2	3.099	0.045	12.32	4.359	0.882	0.013
M3	2.868	0.040	11.44	4.412	0.851	0.012
M4	3.033	0.042	11.881	2.624	0.949	0.013
M5	3.423	0.053	13.675	5.442	0.839	0.015
M6	3.789	0.063	15.252	7.271	0.774	0.018

## 4. Conclusion

pH responsive LSH hydrogels were successfully formulated both with AA and MAA grafting. Formulation A6 and M6 showed maximum drug release. So, they can be a potential pH responsive drug delivery system along with a sustained release action.

## Acknowledgments

F. Shabir gratefully acknowledges financial support provided by the Higher Education Commission (HEC) of Pakistan under ‘HEC Indigenous 5000 fellowship’ program.

## Disclosure statement

No potential conflict of interest was reported by the authors.
